# General Purpose Low-Level Reinforcement Learning Control for Multi-Axis Rotor Aerial Vehicles †

**DOI:** 10.3390/s21134560

**Published:** 2021-07-02

**Authors:** Chen-Huan Pi, Yi-Wei Dai, Kai-Chun Hu, Stone Cheng

**Affiliations:** 1 Department of Mechanical Engineering, National Yang Ming Chiao Tung University, Hsinchu City 30010, Taiwan; john40532.me00@g2.nctu.edu.tw (C.-H.P.); be84n12son25@gmail.com (Y.-W.D.); 2 Department of Applied Mathematics, National Yang Ming Chiao Tung University, Hsinchu City 30010, Taiwan; hu.kaichun@gmail.com

**Keywords:** quadrotor, reinforcement learning, unmanned aerial vehicle

## Abstract

This paper proposes a multipurpose reinforcement learning based low-level multirotor unmanned aerial vehicles control structure constructed using neural networks with model-free training. Other low-level reinforcement learning controllers developed in studies have only been applicable to a model-specific and physical-parameter-specific multirotor, and time-consuming training is required when switching to a different vehicle. We use a 6-degree-of-freedom dynamic model combining acceleration-based control from the policy neural network to overcome these problems. The UAV automatically learns the maneuver by an end-to-end neural network from fusion states to acceleration command. The state estimation is performed using the data from on-board sensors and motion capture. The motion capture system provides spatial position information and a multisensory fusion framework fuses the measurement from the onboard inertia measurement units for compensating the time delay and low update frequency of the capture system. Without requiring expert demonstration, the trained control policy implemented using an improved algorithm can be applied to various multirotors with the output directly mapped to actuators. The algorithm’s ability to control multirotors in the hovering and the tracking task is evaluated. Through simulation and actual experiments, we demonstrate the flight control with a quadrotor and hexrotor by using the trained policy. With the same policy, we verify that we can stabilize the quadrotor and hexrotor in the air under random initial states.

## 1. Introduction

Multirotor vehicle controllers traditionally provide control on two levels. The high-level outer loop provides mission-level control, such as way-point navigation or trajectory planning and tracking. By contrast, the low-level inner loop ensures system stabilization and motion control. When using a control-theoretic-based method for stabilizing a multirotor, parameter tuning [1,2,3] or model identification has often been needed [4,5,6]. Such tuning requires some domain knowledge or the construction of precise kinematical and dynamical models by performing substantial setup and experiments for feedback linearization control [7,8], robust control [9,10], model predictive control [11,12], or sliding mode control (SMC) [13,14,15].

Recently, studies solved many complicated multirotor control problems using reinforcement learning (RL). The RL controller design does not require a predefined controller structure, which limits the regulation or tracking performance of an controller. Greatwood et al. [16] introduced the reinforcement learning that enables autonomous navigation by providing high level path planning decisions for navigation of previously unexplored spaces. The method uses online optimization within a model predictive control (MPC) for navigation and control of quadrotors within a non-convex obstacle field framework, flight test experiments demonstrate within a two dimensional control scenario.

In studies of Hwangbo et al. [17] and Pi et al. [18], they have introduced learning algorithms and implemented a control policy through RL training; this was shown to fully control a quadrotor by using a neural network to perform low-level stabilization and position control directly from the state quadrotor to four motor outputs. In Pi et al. [19], a reinforcement learning with disturbance compensation was proposed using end-to-end manner to compensate wind gusts in outdoor environment. Through introduction of a compensator into the RL control policy, the hovering accuracy and robustness were significantly increased in experiment. These studies obtained results through simulation and in real world environments. Although RL control policies have been hugely successful and obtained results that can compete with those obtained using traditional controllers, the RL controller can only apply the same model as that obtained from the training process, and the multiusability of the trained policy is a problem that remains to be solved. This leads to a neural network control policy that fits only the model-specific quadrotor; another control policy must be created for different quadrotors. The neural network training is time consuming and difficult to perform in general usage situations. To resolve this problem, Wang et al. [20] used a deterministic policy gradient algorithm with integral state to compensate for the varying weight of the quadrotor. Molchanov et al. [21] proposed a method through adjusting the output gain according to the weight of the quadrotor and implemented the method for three quadrotors. The quadrotors remained stable even when their size and weight were different under certain conditions. However, the moment of inertia cannot vary too much in their method, and the command of each motor is still determined directly, meaning that the trained policy can only be applied to one type of multirotor because of the fixed number of rotors during the training process. Dai et al. [22] proposed a control policy which generates moment and force command, which improves the flexibility of the training based RL controller. However, the force and moment control commands depend on the multirotor dynamics in learning process and has difficulties when applied to different vehicles with physical parameters.

Based on these aforementioned studies, we are interested in designing a single RL control policy that can be applied to various multirotor vehicles with arbitrary axis and physical parameters and not require manual tuning to provide stabilization and position control functions. Herein, we propose a controller design method using RL neural network policy with multiusability; the method can resolve the aforementioned RL controller problem noted in previous studies. Multirotor-type UAVs with different weights or different structures and numbers of motors (e.g., quadrotor, hexrotor, and octorotor UAVs) which have differing propeller distributions, and rotation directions can be used to stabilize and exert control through our RL control policy in only a single training session. Such a control structure can be widely used for multirotors of different sizes or several types of vehicle. We demonstrate the recovery and stability performance of the trained control policy obtained using our RL algorithm for a quadrotor and hexrotor, which have different weight and size, both by conducting simulations with arbitrary initial states and real flights. To the best of our knowledge, our work is the first to simulate a low-level attitude-stabilizing RL controller based on multiuse, multirotor neural network policy and demonstrate the feasibility of the controller in real flight.

Section 2 provides a brief introduction of the dynamics of a multirotor. Section 3 introduces the RL theorem and presents our neural structure and control method for multirotors. Section 4 details our experimental results in simulation and real-world experiments. Finally, conclusions are drawn in Section 5.

## 2. Background

In this section, we describe the multirotor dynamic model used in the simulation environment. The multirotor is considered to have a 6-degrees-of-freedom (6DoF) rigid body, which is governed by the Newton–Euler equations. The vehicle translational dynamics are given by Equation (1):

(1) $$\begin{array}{cc} \dot{x} & =v\\ \dot{v} & =m^{-1}\left(Tb_{z}+f_{ext}\right)-g, \end{array}$$

where *m* is the mass of vehicle; x,v and $\dot{v}$ are the position, velocity vectors and acceleration vector, respectively; *T* is the total thrust from the actuators and $f_{e}xt$ is the external disturbance term including aerodynamic drag force in flight. $R=\left[b_{x}\;b_{y}\;b_{z}\right]\in SO\left(3\right)$ is the rotation matrix from the body frame to the inertia reference frame, and $g={\left[0,0,g\right]}^{T}$ is the gravitational acceleration.

The rotational dynamics are given by Equation (2):

(2) $$\begin{array}{cc} \dot{q} & =0.5\cdot q⊗{\left[\begin{array}{c} 0\\ \Omega \end{array}\right]}^{T}\\ \dot{\Omega } & =J^{-1}\left(M-\Omega \times J\Omega \right). \end{array}$$

To prevent gimbal lock in computation, we use a quaternion representation on quadrotor attitude. J is the moment of inertia matrix of the vehicle, $q={\left[q_{0},q_{1},q_{2},q_{3}\right]}^{T}$ is the quaternion vector used to express the orientation of the vehicle, ⊗ is the quaternion multiplication, Ω and $\dot{\Omega }$ are the angular velocity and angular acceleration, and M is the moment generated by the actuators. The rotation transformation between the quaternion *q* to the rotation matrix R can be expressed as Equation (3):

(3) $$\begin{array}{cc} R & =\left[\begin{array}{ccc} 1-2(q_{2}^{2}+q_{3}^{2}) & 2(q_{1}q_{2}-q_{3}q_{0}) & 2(q_{1}q_{3}+q_{2}q_{0})\\ 2(q_{1}q_{2}+q_{3}q_{0}) & 1-2(q_{1}^{2}+q_{3}^{2}) & 2(q_{2}q_{3}-q_{1}q_{0})\\ 2(q_{1}q_{3}-q_{y}q_{0}) & 2(q_{2}q_{3}+q_{x}q_{0}) & 1-2(q_{1}^{2}+q_{2}^{2}) \end{array}\right] \end{array}$$

**Figure 1:**
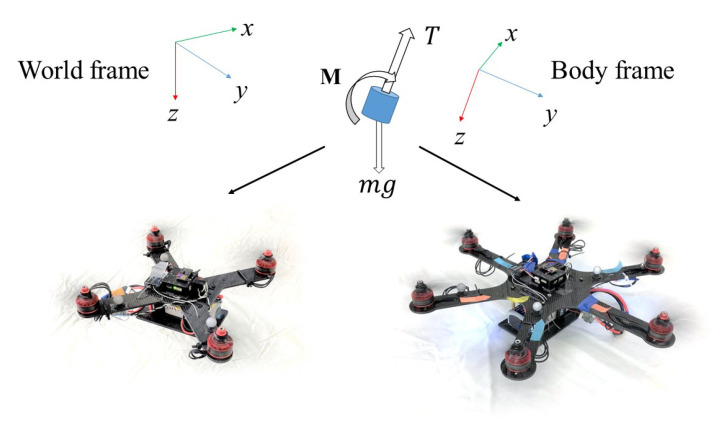
Single trained RL controller from 6DoF model with thrust *T* and moment M to different-configuration multirotors.

The thrust generated from motors are assumed to be aligned to the z-axis of the multirotor. External disturbance and drag force are neglected on the dynamic model of the vehicle in simulation environment in training process and regard as the uncertainties to the control policy. For different multirotors, the mapping from thrust *T* to the moment is different and dependent on the placement of the rotors. Figure 1 shows the two types of multirotor, the quadrotor and hexrotor, that were used in our real world experiment. They have the same thrust force direction applied to the body frame *z*-axis but different moment from each rotor.

## 3. Reinforcement Learning Algorithm and Implementation

In this section, we present our policy training method and the structure of the experiment verification for different multi-axis rotors. The experimental results are discussed in Section 4.

### 3.1. Algorithm

The RL is usually implemented in conjunction with the a Markov decision process (MDP). The MDP is defined by the tuple (S,A,P,r), where S is the state space, A is the action space, $P:S\times A\times S\to R^{+}$ is the state transition probability, and r:S×A→R is the reward function. The goal of the MDP is to determine the optimal decision, whereas the purpose of RL is to solve the MDP problem through a learning process and propose the optimal policy $\pi :S\times A\to R^{+}$.

The value function indicates the performance of a policy by the total expected reward with a discounted factor γ∈(0,1) and was written as Equation (4):

(4) $$V^{\pi }\left(s\right)=E\left[\sum_{t\geq 0}\gamma^{t}r_{t}|s_{0}=s,a∼\pi \right],$$

and the learning process involves increasing it to as large as possible. To update to a better policy π, we start from the equation of the difference between policy π and another policy μ:

(5) $$V^{\pi }\left(s\right)-V^{\mu }\left(s\right)=\sum_{x\in S}\rho_{s}^{\pi }\left(x\right)\sum_{a\in A}\left[\pi (a|x)-\mu (a|x)\right]A^{\mu }\left(x,a\right),$$

where $\rho_{s}^{\pi }\left(x\right)$ is the state visited frequency with discount, $A^{\mu }\left(s,a\right)$ is the advantage, $Q^{\mu }\left(s,a\right)$ is the the state-action value and can be written as following equations respectively.

(6) $$\rho_{s}^{\pi }\left(x\right)=\sum_{t\geq 0}\gamma^{t}P\left(s_{t}=x|s_{0}=s,a∼\pi \right)$$

(7) $$A^{\mu }\left(s,a\right)=Q^{\mu }\left(s,a\right)-V^{\mu }\left(s\right)$$

(8) $$Q^{\mu }\left(s,a\right)=E\left[r_{t}+\gamma V^{\mu }\left(s_{t+1}\right)|s_{t}=s,a_{t}=a\right]$$

According to Equation (5), it shows that if Equation (9) holds

(9) $$\left[\pi (a|x)-\mu (a|x)\right]A^{\mu }\left(x,a\right)\geq 0$$

implies the Equation (10):

(10) $$V^{\pi }\left(s\right)\geq V^{\mu }\left(s\right),$$

and this indicates that the policy π is better than policy μ on state *s*. Equation (5) shows that a better policy can be constructed using Equation (9) by adjusting the probability of a specific state-action pair according to the advantage function (7).

### 3.2. Implementation

The relation between Equations (9) and (10) implies that given a policy μ, a better policy π can be constructed by satisfying the inequality of Equation (9). Therefore, the proper estimation of $A^{\mu }$ in Equation (9) is the major topic and the approach in this work is based on [18].

In the typical model-free RL approaches, the value estimation is based on minimizing the squared Bellman error as Equation (11):

(11) $$L\left[\hat{V}\right]=E[|r_{t}+\gamma \hat{V}\left(s_{t+1}\right)-\hat{V}\left(s_{t}\right){|}^{2}\left|\left(s_{t},a_{t}\right)∼\mu \right],$$

where $\hat{V}$ is an approximated value function $V^{\mu }$. This approach is known as temporal difference learning [23]. In this study, we use a variance temporal difference learning method, or called V-trace [24] to replace the fitting target $r_{t}+\gamma V^{\mu }\left(s_{t+1}\right)$ in Equation (11). The value and advantage function can be rewritten as following equations,

(12) $$V_{t}^{trace}=\hat{V}\left(s_{t}\right)+\min\left(1,\frac{\pi (a_{t}|s_{t})}{\mu (a_{t}|s_{t})}\right)A_{t}^{trace}$$

(13) $$A_{t}^{trace}=A_{t}+\gamma \min\left(1,\frac{\pi (a_{t+1}|s_{t+1})}{\mu (a_{t+1}|s_{t+1})}\right)A_{t+1}^{trace},$$

(14) $$A_{t}=r_{t}+\hat{V}\left(s_{t+1}\right)-\hat{V}\left(s_{t}\right).$$

Therefore, the objective in our value fitting is to minimize Equation (15):

(15) $$L_{value}=E\left[{\left(V_{t}^{trace}-\hat{V}\left(s_{t}\right)\right)}^{2}\right].$$

For improving the policy, an ideal approach is to construct a new policy π that satisfies Equation (9). However, without the preliminary of the model, there is no efficient way to do so. On the other hand, in the context of machine learning, this problem is treated by empirical risk minimization. For instance, we minimize Equation (16):

(16) $$L_{policy}=\sum_{(s,a)\in B}\max\left\{0,\epsilon -A^{trace}\left(s,a\right)\log\frac{\pi (a|s)}{\mu (a|s)}\right\},$$

where ϵ>0 is a predefined parameter called margin, B is the buffer that stores the sampled transition. The loss in Equation (16) comes from the state-action pairs that failed to satisfy Equation (9) in B. We optimize both Equations (11) and (16) by a variance stochastic gradient descent, Adam [25].

In order to calculate Equation (12), the state-action pairs need to be a time sequence (trajectory) thus we solve Equations (1) and (2) by numerical integration, i.e., Euler method, in *N* steps. In each step n∈[0,N−1], we sample single action from the current policy and estimate the next state every simulated duration 0.01 s. For the case of n=0, the state is generated by random sampling from the interesting control region. In practice, we set this region by a square space in position, velocity, angular velocity, and arbitrary unit quaternion in orientation. The state $s_{t}$ and action $a_{t}$ in Equation (12) are represented as following equations,

(17) $$\begin{array}{cc} s_{t}= & {\left(\begin{array}{cccc} x & v & q & \Omega \end{array}\right)}_{t}^{T},\\ a_{t}= & {\left(\dot{\Omega },\dot{v}\right)}_{t}. \end{array}$$

The state transition is determined by Equation (18):

(18) $${\left[\begin{array}{c} x\\ v\\ q\\ \Omega \end{array}\right]}_{t+1}={\left[\begin{array}{c} x\\ v\\ q\\ \Omega \end{array}\right]}_{t}+\left[\begin{array}{c} v_{t}\\ m^{-1}Tb_{z}-g\\ 0.5\cdot q_{t}⊗\left[\begin{array}{c} 0\\ \Omega_{t} \end{array}\right]\\ J^{-1}\left(M-\Omega_{t}\times J\Omega_{t}\right) \end{array}\right]\Delta t$$

(19) $$\begin{array}{c} \left[\begin{array}{c} M\\ T \end{array}\right]=\left[\begin{array}{cc} J & 0\\ 0 & m \end{array}\right]a+\left[\begin{array}{c} \Omega \times J\Omega \\ 0 \end{array}\right]-\left[\begin{array}{c} 0\\ g \end{array}\right] \end{array}$$

The outputs of policy function π are the mean α and standard deviation σ of the Gaussian distribution, which is defined as the probability density function of the normalized angular and translational acceleration generated by the rotors. The π function can be written as Equation (20):

(20) $$\pi \left(a|s\right)=\frac{1}{\sqrt{2\pi \sigma^{2}\left(s\right)}}e^{-\frac{{\left(a-\alpha \left(s\right)\right)}^{2}}{2\sigma^{2}\left(s\right)}}.$$

The gravitational acceleration *g* is added and considered as the feed-forward term to directly compensate for take-off weight.

We store all trajectories into the memory buffer B with fixed size. Once the buffer is filled, the oldest data are removed, freeing space for new data. The training samples for optimizing Equations (15) and (16) are sampled from this buffer. The Algorithm 1 presents the pseudo code of RL agent training.
**Algorithm 1** Learning Algorithm1:Initialize policy and value function parameters $\theta_{\pi },\theta_{V}$2:Set the maximum episode *N* and maximum step *T*3:**repeat**4: **for** *i* in [0,N−1] **do**5:  Randomly initialize the states $s_{0}$ of vehicle6:  **for** *t* in [0,T−1] **do**7:   Making a decision $a_{t}$ according to $\pi (a|s_{t})$8:   Evaluate $s_{t+1}$ according to (18)9:   Collect $D_{t}=\left\{s_{t},a_{t},\pi \left(a_{t}|s_{t}\right),r_{t},s_{t+1}\right\}$10:  Save trajectory $\tau_{i}=\left\{D_{0},D_{1},\ldots ,D_{T-1}\right\}$ to memory buffer B11: Randomly sample *M* trajectories $\left\{\tau \right\}$ from B12: **for** τ in $\left\{\tau \right\}$ **do**13:  set tmp=014:  **for** j=T−1 to 0 **do**15:   $Q_{j}=r_{j}+\gamma \hat{V}\left(s_{j+1}\right)$16:   $A_{j}=Q_{j}-\hat{V}\left(s_{j}\right)$17:   $A_{j}^{trace}=A_{j}+\gamma \times tmp$18:   $tmp=\min\left(1,\frac{\pi (a_{t}|s_{t};\theta )}{\pi (a_{t}|s_{t};\theta_{old})}\right)A_{j}^{trace}$19:   $V_{j}^{trace}=\hat{V_{j}}+tmp$20:  ${\hat{g}}_{policy}=\frac{1}{T}\sum_{j=0}^{T-1}\nabla L_{policy,j}$21:  Update $\theta_{\pi }$ using Adam optimizer by ${\hat{g}}_{policy}$22:  ${\hat{g}}_{value}=\frac{1}{T}\sum_{j=0}^{T-1}\nabla L_{value,j}$23:  Update $\theta_{V}$ using Adam optimizer by ${\hat{g}}_{value}$24:**until** training success

The neural networks are composed of artificial neuron node layers, and each layer processes affine transformation and non-linear mapping by using an activation function, expressed as Equation (21):

(21) $$\begin{array}{cc} \; & z^{\left[i\right]}=W^{\left[i\right]}a^{\left[i-1\right]}+b^{\left[i\right]}\\ \; & a^{\left[i\right]}=\kappa \left(z^{\left[i\right]}\right) \end{array}$$

where $a^{\left[i\right]}$ is the output of layer *i*; $a^{\left[i-1\right]}$ is the input (vehicle state or output from previous layer); W,b∈θ are the weight and bias of the neural network, respectively; and κ is the activation function.

**Figure 2:**
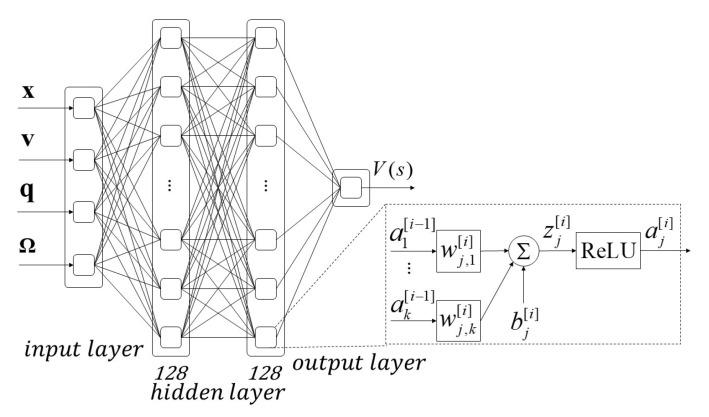
Value function using neural network approximation; the function consists of two hidden layers with 128 rectified linear unit nodes in each layer. The input is the state of vehicle, where x,v,q and Ω are the position, velocity, quaternion, and angular velocity, and the output is the estimated value of state V(s).

**Figure 3:**
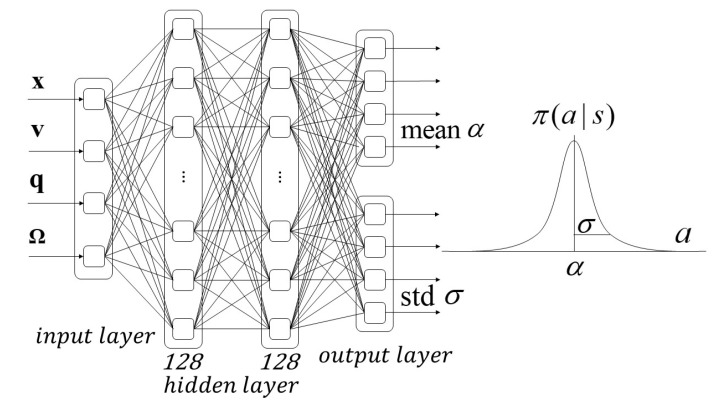
Policy neural network approximation structure, consisting of two hidden layers with 32 rectified linear unit nodes in each layer. The neural network outputs the mean α and standard deviation σ of the Gaussian distribution to generate the control command.

In this paper, we have two neural networks: one used for state value estimation and another for determining the actions, as illustrated in Figure 2 and Figure 3. The value function is used for neural network approximation; the function consists of two hidden layers with 128 nodes in hidden layer. Rectified linear unit activation function is used in hidden layer and output layer. The inputs of the function are the states of vehicle (position, velocity, quaternion, and angular velocity), and the output is the estimated value of state V(s). The policy neural network approximation structure consists of two hidden layers with 32 rectified linear unit nodes in each layer. The policy neural network outputs the mean α and standard deviation σ of the Gaussian distribution to generate the control command. The sinusoidal function is used as the activation function in the output layer to ensure the output is bounded, and scales are applied to delimit the desired acceleration range.

## 4. Experiments and Verification

**Figure 4:**
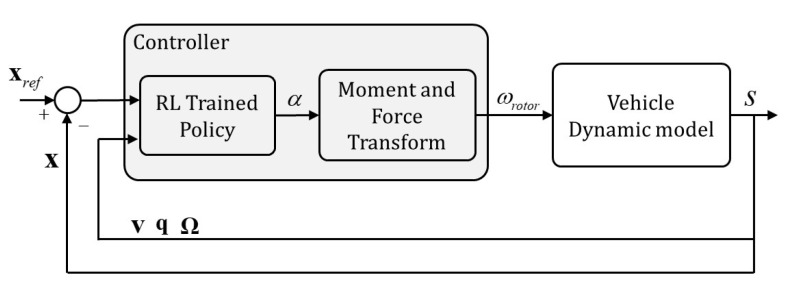
Closed-loop control system for a multirotor UAV, where α is the mean output, *M* is the total moment, *T* is the total thrust, and *s* is the vehicle’s state.

In this section, we present our proposed control structure and training method for control policy. After the policy is successfully trained and verified in the simulation environment, the control policy then is applied to multiple types of multirotor. In our experiment, we use a quadrotor and hexrotor with differing physical parameters (detailed in Table 1) and test them in both simulation and real world environments by using the control closed-loop structure presented in Figure 4.

### 4.1. Reinforcement Learning Training

On the basis of the algorithms discussed in Section 3, we define our value network to have two layers and 128 nodes in each layer. The policy network also uses two layers but has 32 nodes in each layer to shorten the computation time when applied onto the on-board flight computer. The Tensorflow framework [26] is used for implementing the RL training algorithm described in Section 3 under the Python environment. The version of Tensorflow is r1.13. Both the neural networks take the states of the 6DoF rigid body (position, velocity, quaternion of rotation, and angular velocity) and the control policy π(a|s) outputs angular and translational acceleration command. The output command is the translational and rotational acceleration of the multirotor and is limited as $\dot{v}\in \left[-40,9.8\right]$ m/s${}^{2}$ and $\dot{\Omega }\in \left[-100,100\right]$ rad/s${}^{2}$. The maximum translational acceleration cannot exceed 9.8 m/s${}^{2}$ due to the rotor can only generate negative direction thrust force on body *z*-axis, while the acceleration on the positive direction can only given by the gravity.

**Figure 5:**
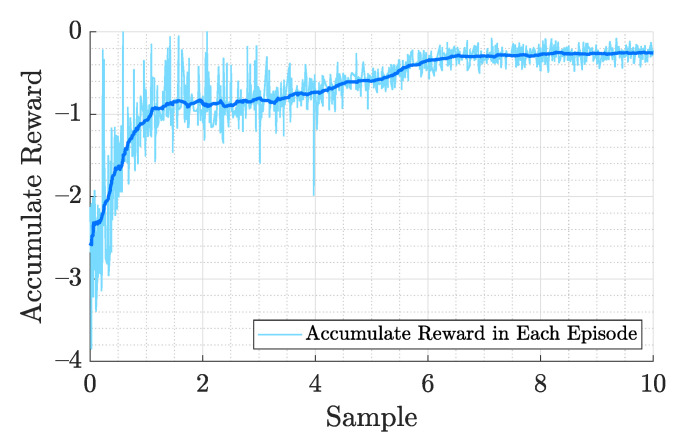
Accumulated reward training curve of each episode in RL controller optimization and is sampled every 100 steps update. The solid line indicates the average value. The training takes approximately 10 million steps to train a control policy that achieves steady hovering at a designated position.

**Figure 6:**
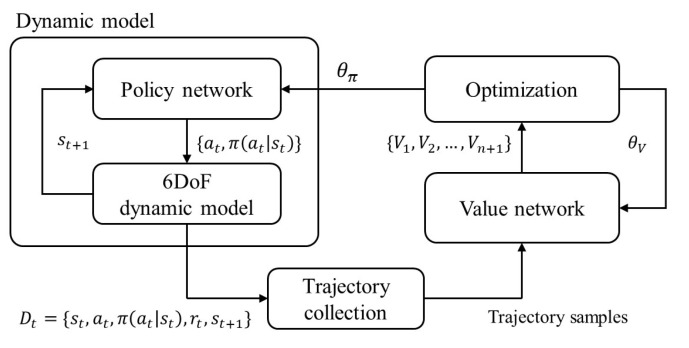
Block diagram of the training process. The $D_{t}$ are the data generated by the dynamic model which include the state of the dynamic model $s_{t}$, action $a_{t}$ determined by the policy function, the policy probability $\pi (a_{t}|s_{t})$, and the reward $r_{t}$. The generated data are collected as the trajectory sample and used in the optimization algorithm.

For reinforcement policy training, we design the reward function as following equations:

(22) $$\begin{array}{c} \mathrm{reward}=-\left[\begin{array}{ccc} w_{1} & w_{2} & w_{3} \end{array}\right]{\left[\begin{array}{ccc} \|q_{e}\| & \|p_{e}\| & \left\|\Omega \right\| \end{array}\right]}^{T}, \end{array}$$

(23) $$q_{e}=q_{d}q^{-1}$$

(24) $$p_{e}=x_{d}-x$$

where $q_{d}$, $x_{d}$, and Ω are the desired quaternion, desired position, and angular velocity, respectively, and $w_{1}$ to $w_{3}$ are the weights of these errors which are all set to 0.002. The quantities of $w_{1}$ and $w_{2}$ need greater than zero while the magnitude determines the priority of reducing position error and heading error. The number $w_{3}$ penalizes the angular velocity. A smaller $w_{3}$ implies a more aggressive control policy. We analyzed the necessity of different items in the RL training reward function (22) to simulate the flight performance of the quadrotor. We are interested in simpler reward function with fewer hyperparameters. The reward is normalized into [0,−1] to ensure convergence speed. The training takes approximately 10 million steps to train a control policy that achieves steady hovering at a designated position and the training curve of accumulate reward is shown in Figure 5. The training progress of the neural network navigates the vehicle to the designed target, and the accumulated rewards come to stable after around 70,000 episodes. In the following 30,000 episodes, the evaluation of reward does not show any significant progress, and the training can be stopped at this time. Figure 6 shows the block diagram of the training process. The $D_{t}$ are the data generated by the dynamic model which include the state of the dynamic model $s_{t},s_{t+1}$, action $a_{t}$ determined by the policy function, the policy probability $\pi (a_{t}|s_{t})$, and the reward $r_{t}$. The generated data are collected as the trajectory sample in the memory buffer and used in the optimization algorithm to update the weight of policy and value network $\theta_{\pi },\theta_{V}$.

Despite the fact that RL is a model free learning algorithm, sampling the training batch episodes from the real multirotor is impractical. The multirotor is an unstable system without proper controller. The multirotor would fail too many times before the RL control policy learned to maintain its flight. The whole training process is completed in a simulation environment.

### 4.2. Mapping Strategy

For implementing our trained policy with various vehicles, we apply the closed-loop system 6DoF dynamic model presented in Figure 4 to the quadrotor and hexrotor described in Table 1. The RL trained policy generates the translational and rotational control acceleration command with inputs of vehicle position, velocity, rotation, and angular velocity. Then, the outputs from the control policy are converted to the rotation speed $\omega_{rotor}$ of each rotor in each vehicle. The thrust force from the rotor speed is given as Equation (25),

(25) $$\begin{array}{cc} \; & M_{rotor\;z}=c_{d}\omega_{rotor}^{2}+J_{rotor}{\dot{\omega }}_{rotor}\\ \; & T_{rotor}=c_{f}\omega_{rotor}^{2} \end{array}$$

where and $c_{f}$, $c_{d}$ are the coefficients of the generated force and *z*-axis moment, respectively. The $J_{rotor}$ is the rotor and propeller moment of inertia. We only consider the drag force term on *z*-axis moment in the training process due to the $I_{rotor}{\dot{\omega }}_{rotor}$ is relative small.

For the quadrotor model, Equation (26) gives the conversion from the propeller rotation speed of quadrotor $\omega_{quad}=\left[\omega_{1},\omega_{2},\omega_{3},\omega_{4}\right]$ and hexrotor $\omega_{hex}=\left[\omega_{1},\omega_{2},\omega_{3},\omega_{4},\omega_{5},\omega_{6}\right]$ to force *T* and moment M. The ${\omega_{quad}}^{2}$ and ${\omega_{hex}}^{2}$ represent the element-wise square of the vector.

(26) $$\left[\begin{array}{c} M\\ T \end{array}\right]=\left[\begin{array}{cccc} -lc_{f} & lc_{f} & lc_{f} & -lc_{f}\\ lc_{f} & -lc_{f} & lc_{f} & -lc_{f}\\ c_{d} & c_{d} & -c_{d} & -c_{d}\\ c_{f} & c_{f} & c_{f} & c_{f} \end{array}\right]{\omega_{quad}}^{2}$$

The hexrotor conversion would be Equation (27):

(27) $$\left[\begin{array}{c} M\\ T \end{array}\right]=\left[\begin{array}{ccccccc} -lc_{f} & lc_{f} & lc_{f}/2 & -lc_{f}/2 & -lc_{f}/2 & lc_{f}/2\\ 0 & 0 & \sqrt{3}lc_{f}/2 & -\sqrt{3}lc_{f}/2 & \sqrt{3}lc_{f}/2 & \sqrt{3}lc_{f}/2\\ -c_{d} & c_{d} & -c_{d} & c_{d} & c_{d} & -c_{d} & \\ c_{f} & c_{f} & c_{f} & c_{f} & c_{f} & c_{f} \end{array}\right]{\omega_{hex}}^{2}.$$

where *l* is the arm length of the vehicle.

However, to calculate the inverse transformation from force *T* and moment M to motor thrust for the hexrotor, we add two additional restrictions as Equation (28) for solving the solution of $\omega_{hex}$:

(28) $$\begin{array}{c} \left[\begin{array}{c} 0\\ 0 \end{array}\right]=\left[\begin{array}{cccccc} c_{f} & c_{f} & -c_{f} & -c_{f} & 0 & 0\\ 0 & 0 & c_{f} & c_{f} & -c_{f} & -c_{f} \end{array}\right]{\omega_{hex}}^{2} \end{array}$$

**Figure 7:**
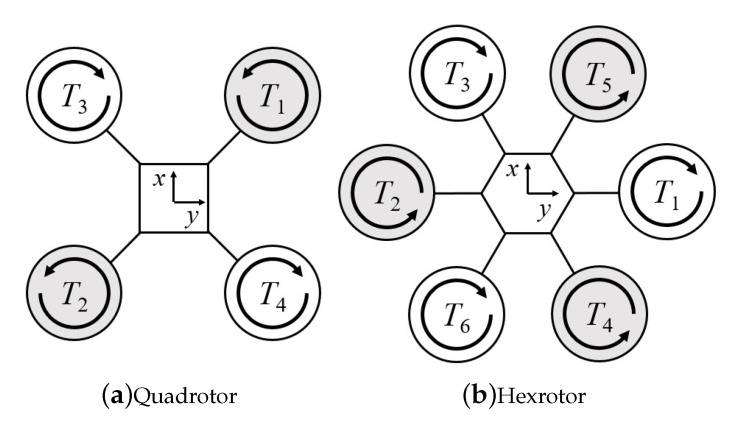
Top-down view of (**a**) quadrotor and (**b**) hexrotor models with propeller thrust pairs $\left[\left(\omega_{1},\omega_{2}\right),\left(\omega_{3},\omega_{4}\right),\left(\omega_{5},\omega_{6}\right)\right]$. Motors are indexed in the opposite direction starting from the $T_{1}$ motor, which generates thrust force $T_{1}$.

We design these additional constraints for the hexrotor to divide the six rotors into three pairs, namely $\left[\left(\omega_{1},\omega_{2}\right),\left(\omega_{3},\omega_{4}\right),\left(\omega_{5},\omega_{6}\right)\right]\in \omega_{hex}$, as illustrated in Figure 7. This ensures that each motor pair generates equal thrust. The constraints distribute the desired total thrust to each motor evenly and prevent the saturation of motor thrust.

### 4.3. Simulation Verification

To verify the result of control policy training on the multirotor, the controller is tested in the simulation environment. We create the dynamic models according to Equations (1) and (2), and the physical parameters are presented in Table 1. The hexrotor has 50% more weight and 3 times more moment of inertia than the quadrotor to verify the control strategy applied on different vehicles. Notice that the rotor dynamics is not included in the dynamic model of simulation for RL control agent training. To verify that our proposed RL policy controller structure could be implemented with multirotors, we conduct 20 flight tests starting from arbitrary initial conditions in a 4 m × 4 m × 4 m space, velocity, and angular velocity between ±1m/s and ±1rad/s with both the quadrotor and hexrotor vehicles detailed in Table 1 for the simulation environment.

**Figure 8:**
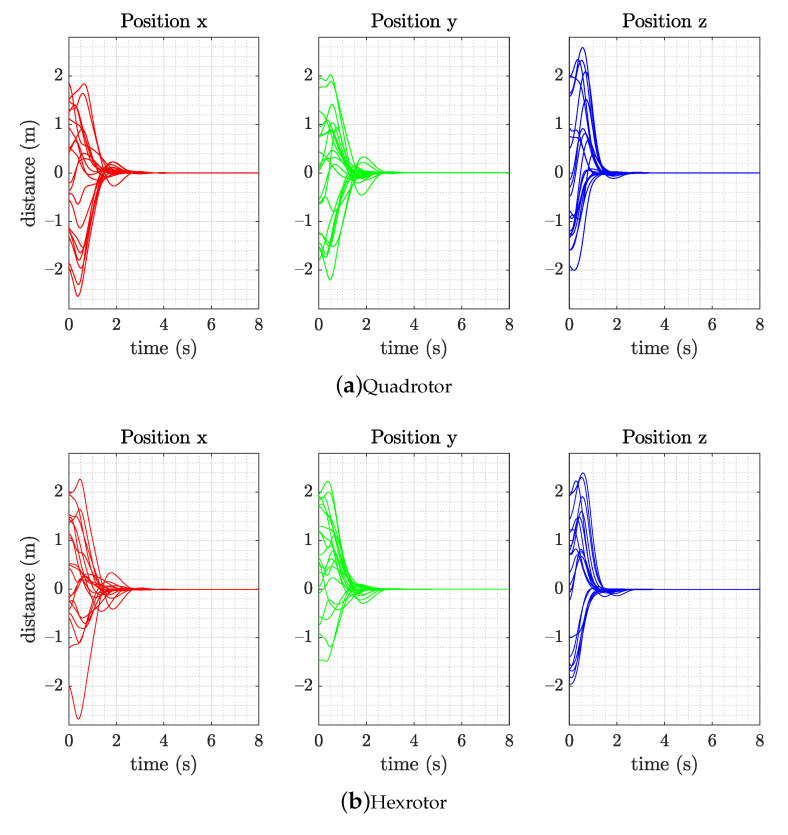
Simulation response of the (**a**) quadrotor and (**b**) hexrotor models, which achieved hovering capability within 8 s with 20 random initial states in the simulation flight test (position, velocity, attitude, and angular velocity).

Here, we combine sensors and thrust noise to hover the quadrotor at a fixed point, record its attitude for 60 s, and report the RMS position error. When applying the RL controller from the training section to the multirotors, the trained control policy network is extracted and only the mean of the policy action (translational and rotational acceleration) α is chosen as the output command to the mapping strategy without using the randomization from standard deviation σ. Figure 8 illustrates the flight results of the simulation. The vehicles return to their point of origin and hover successfully in all randomly initialized hovering trials.

### 4.4. Real-World Verification

**Figure 9:**
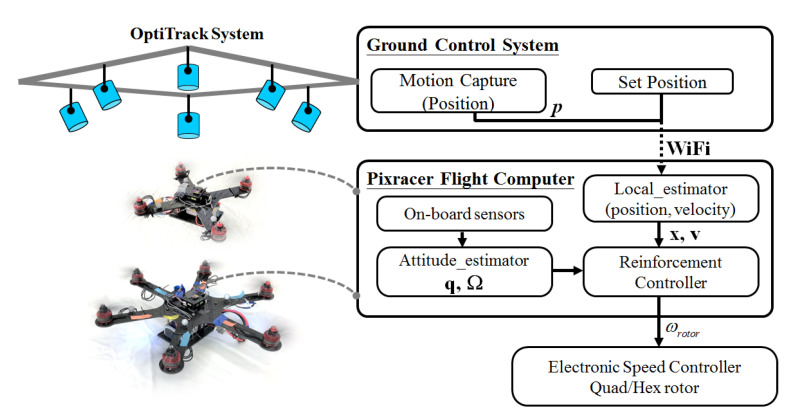
The motion capture framework (OptiTrack Prime 13, 1.3 MP Resolution, 240 FPS) for real-world verification is exploited to interface with the vehicles. The trained policy and control structure are implemented using Pixracer flight controller and WiFi connection to achieve position feedback with wireless communication.

After the vehicle showed successful flight in the simulator, we transfer our algorithm to a real world flight test. In this test, our trained policy and control structure are implemented using a Pixracer flight control board (mRobotics, Chula Vista, CA, USA), which has an STM32F427 micro-controller clocking at 180 MHz and WiFi connection ability for wireless communication, enabling motion capture position feedback. The state estimation is performed using the on-board sensor and motion capture with extended Kalman filter. The on-board computation is conducted at 250 Hz, whereas the feedback from the OptiTrack motion capture system is provided at 120 Hz 3D precision in 0.5 mm. Consumer-grade standard parts (motors and propellers) are selected when building the multirotors. Each propeller can generate a maximum of 8 N. A consumer-grade electronic speed controller (ESC) mapping function of speed command to rotor thrust is built through experiment measurement. Figure 9 shows the system diagram used for the experiments. The *p* denote the desired goal, x,v is the estimated position and velocity from local estimator. Quaternion q, and angular velocity Ω in body frame represent measurements from flight controller on-board sensors. $\omega_{rotor}$ is thrust force in newton and rotor speed in rad/s.

**Figure 10:**
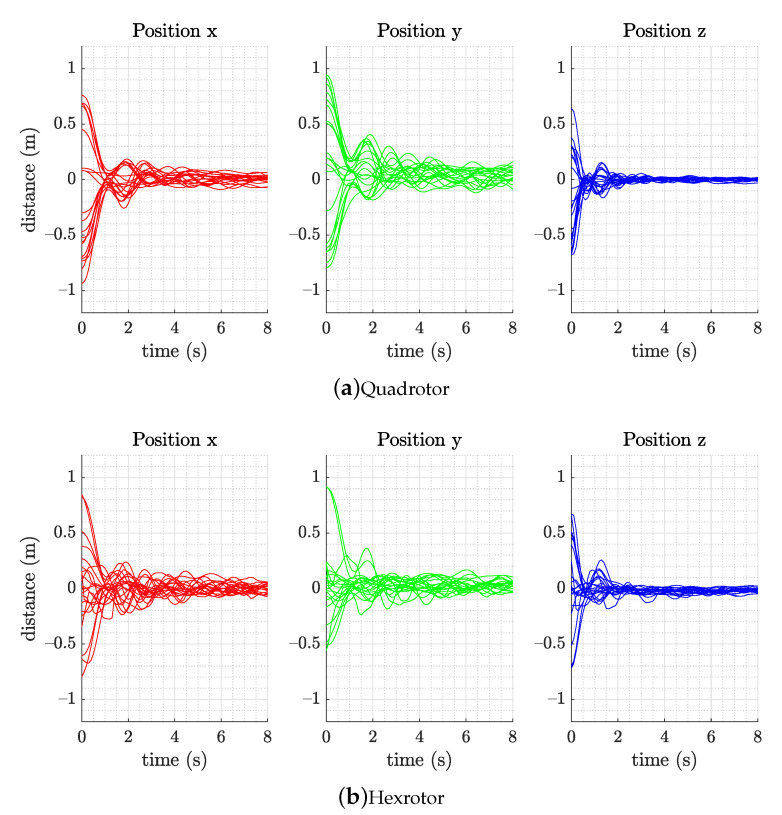
Experimental result of the (**a**) quadrotor and (**b**) hexrotor models, which achieved hovering capability within 8 s with 20 random initial states in the simulation flight test (position, velocity, attitude, and angular velocity).

**Figure 11:**
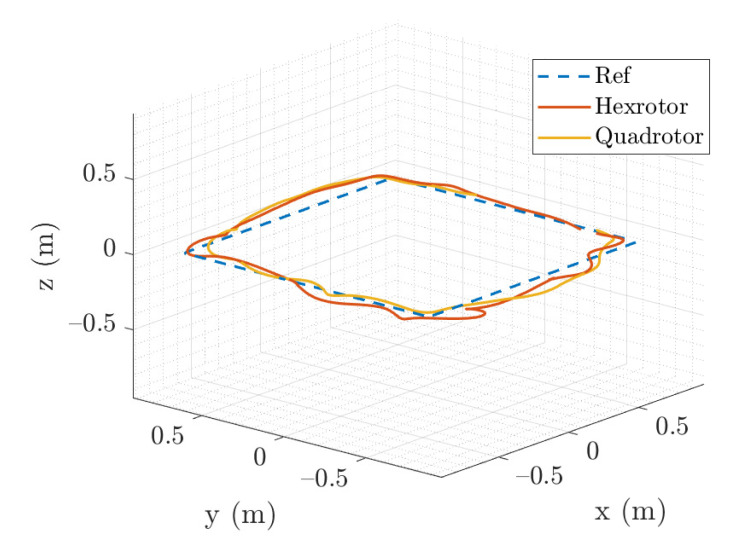
The quadrotor and hexrotor track a 1.5-m-wide square path with waypoint moving in 0.3 m/s.

In the RL controller validation experiment, we perform three experiment to assess the RL performance. (1) A remotely piloted task is performed, starting from 20 random initial positions in a 2 m × 2 m × 2 m space, velocity, and angular velocity between ±0.2 m/s and ±0.4 rad/s. The vehicle is set to fly back and hover at its point of origin. A smaller site is used because of the limitation of the environment. The results of this experiment are illustrated in Figure 10. The three-dimensional position plot presents the hovering positioning performance of both the quadrotor and hexrotor. (2) We analyze the steady state hovering performance calculating the root mean square error (RMSE) on each axis for vehicles in one minute and are listed in Table 2. The z-RMSE on hexrotor is two times larger than the quadrotor, especially in the steady hovering which can be observed on Figure 10. We believe this is due to the imperfection of the rotor thrust mapping function. (3) We also demonstrate the way-point following capability of the RL controller and compare the behavior and flying trajectory of the two vehicles. Figure 11 shows that the quadrotor and hexrotor track a 1.5-m-wide square path with velocity command 0.3 m/s. The tracking RMS error is 0.11 m and 0.16 m of quadrotor and hexrotor separately. The figure shows the two vehicles have similar behavior and trajectory in spite of having different structure and physical parameters.

In the real world flight test, a back and forth movement through which the vehicles approach a set point on the x,y axes is observed before they reach their point of origin, unlike in the simulations. First, we attribute this to the imperfect inverse transform between the desired thrust and ESC command. In the simulator training process, only gravity and the forces generated by the motors were considered to construct the simplest dynamic model. We do not employ motor speed as feedback or identify motor dynamics when implement to real multirotors. How the ESC regulates the motor speed is unknown. This unknown gives an inaccurate angular acceleration to the frame and affect the transient movement of multirotors. Second, the simulation environment does not include the measurement noise of on-board sensors and the communication delay in sensor feedback, especially in wireless communication on motion capture position feedback that influence the controller performance. However, the experimental results indicate that our RL controller structure can nonetheless stabilize the multirotors and regulate the set point.

## 5. Conclusions

In this article, we proposed a RL controller design method in which a well-trained control strategy can be applied to UAVs with varying physical parameters or even of different types. Starting from constructing a 6DoF rigid body model and used in the RL training. The trained control policy can be applied to different types of multirotors through the mapping strategy without manual parameters tuning. The method’s adaptability for multirotor UAVs is demonstrated. By contrast, the trained RL controllers presented in [17,18,21] can only be used for a specific multirotor with the same physical structure and parameters. In our method, the policy neural network output can be converted for each actuator unit according to the dynamic model of various geometric characteristics of the vehicle. Furthermore, the proposed strategy keeps the advantage of high robustness to harsh initial conditions as in previous studies of RL quadrotor controllers.

The simulation and experimental results show that our controller works for both a quadrotor and hexrotor when starting from randomly initialized states (position, velocity, orientation, and angular velocity) in a 2-m-wide cubic space. The hexrotor has 50% more weight and 3 times more moment of inertia than the quadrotor. Our method demonstrates the control policy can be applied onto the vehicles with wide derivation physical parameters using a single trained controller. The waypoint following experiment also shows that the two vehicles have similar behavior and flight trajectory in spite of having different structure and physical parameters. Through a single RL policy function from training, the controller stabilizes the attitude of the vehicle and regulates the set position successfully. The experiments also demonstrate the robustness of the RL controller to the unknown dynamics of motor ESC and the latency of the sensors feedback especially in motion capture wireless communication, which are not included in the RL control policy training process. Our method opens wide possibilities for directly applying the RL controller to various custom-built multirotors without the need to perform a redundant training process.

The applications for the urban flight application, the wind gust is the first challenge that would affect the flight performance. A disturbance compensator can be combined with the RL controller to reduce the wind gust effect and improve the flight performance. Another work would be improving the mapping for actuators output. The RL controller was trained under the constraint of maximum translational and rotational acceleration to simulate the thrust limitation of rotors that can generate. The rotors thrust commands may have better solution using the mapping strategy for better application requirement, which would be another topic to discuss in future work.

## Figures and Tables

**Table 1:** Physical properties of vehicles. Diag(a) is the 3×3 diagonal matrix with entries that are the three elements of vector *a*.

	Quadrotor	Hexrotor
Mass	0.673	1.089
(kg)		
Inertia	diag(27,33,52)	diag(83,91,151)
(kgcm${}^{2}$)		
Arm Length (m)	0.126	0.180
Rotor Diameter (m)	0.127	0.127

**Table 2:** Root mean square error of 1 min hovering of quadrotor and hexrotor at the point of origin.

	*x*-RMSE	*y*-RMSE	*z*-RMSE
	(cm)	(cm)	(cm)
Quadrotor	3.61	5.41	1.05
Hexrotor	2.82	4.17	1.95

## Data Availability

The data presented in this study are available on request from the corresponding author.
